# Beyond the Mortality Effect: *Spodoptera frugiperda* Multiple Nucleopolyhedrovirus Promotes Changes in Feeding and Inhibits Larval Growth and Weight Gain in Fall Armyworm

**DOI:** 10.3390/microorganisms14010001

**Published:** 2025-12-19

**Authors:** Bianca Marina Costa Nascimento, Anderson Delfino Mauricio Nunes, Silvio Lisboa de Souza Junior, Luiz Fernando de Santana Santos, Fabio Mielezrski, Carlos Henrique de Brito, Breno Álef Parnaíba Cândido, Isabel Lopes de Medeiros, Wanderlan Gonçalves Praxedes Júnior, Janayne Maria Rezende, Francisco de Sousa Ramalho, Rosilda Mara Mussury Franco Silva, José Bruno Malaquias

**Affiliations:** 1 Graduate Program in Entomology, Universidade de São Paulo, Campus de Ribeirão Preto, Av. Bandeirantes 3900-Vila Monte Alegre, Ribeirão Preto 14040-900, SP, Brazil; 2 Department of Crop Production and Environmental Sciences, Federal University of Paraiba, Campus II, Rodovia BR 079, km 12, Areia 58397-000, PB, Brazil; 3 Mediterranean Institute for Agriculture, Environment and Development, 7002-554 Evora, Portugal; 4 AgBiTech, Rod. R-2, 3.061-Chácaras Califórnia, Goiânia 74690-631, GO, Brazil; 5 Embrapa Algodão, Rua Osvaldo Cruz, Campina Grande 58428-095, PB, Brazil; 6 Faculty of Biological and Environmental Sciences, Federal University of Grande Dourados, Dourados Highway-Itahum, km 12, Dourados 79804-970, MS, Brazil

**Keywords:** maize, sublethal, infection, Baculoviridae

## Abstract

Infection by *Spodoptera frugiperda* multiple nucleopolyhedrovirus (SfMNPV) profoundly alters the physiology of *S. frugiperda* (Lepidoptera: Noctuidae), promoting molecular responses that activate genes related to cellular defense. These responses demand substantial energy and lead to feeding dysfunction. Knowledge about the effects of SfMNPV on weight gain and leaf consumption rate is still incipient; in this context, we evaluated the survival rate, weight gain, leaf consumption rate, and body size of *S. frugiperda* exposed to different concentrations of SfMNPV. A completely randomized design was used in the laboratory. Treatments consisted of SfMNPV from the commercial product Cartugen^®^, diluted at different concentrations and applied on a Petri dish: 9.00 × 10^3^, 1.80 × 10^4^, 3.75 × 10^4^, 7.50 × 10^4^, 1.35 × 10^5^, 2.25 × 10^5^, and 3.75 × 10^5^ occlusion bodies (OBs)/mL. Fifty first-instar larvae were used per treatment. The median lethal concentration was estimated at 1.32 × 10^5^ OBs/mL (95% confidence interval = 1.22 × 10^5^–1.43 × 10^5^ OBs/mL). Nonlinear regression analysis of sublethal effects showed that the expected weight of the control (W_0_), the angular parameter (B), and the effective concentration capable of reducing larval weight by 50% (EC_50_) were 47.40 mg, 1.42, and 1.16 × 10^4^ OBs/mL, respectively. Leaf consumption was inhibited at lower concentrations and increased at higher concentrations among surviving larvae, and larval growth (measured by head diameter, body length, and body width) was lower when larvae were exposed to SfMNPV than in the control. Our data suggest that SfMNPV at low concentrations causes dysfunctions that prevent normal development in surviving individuals, resulting in reduced consumption rate, body growth, and weight gain. Overall, our analysis indicates that the impact of SfMNPV extends beyond mortality; low concentrations can affect larval growth and feeding consumption rate in *S. frugiperda*.

## 1. Introduction

*Spodoptera frugiperda* (JE Smith) (Lepidoptera: Noctuidae), commonly known as the fall armyworm, is a polyphagous pest native to the Americas that has recently spread to other regions of the world, including Africa, Asia, and Oceania [1]. This pest can attack more than 350 plant species, with host records from 76 plant families [principally Poaceae (106 species), Asteraceae (31 species), and Fabaceae (31 species)], and corn (*Zea mays* L.) is considered its primary host [2,3]. The larvae feed on leaves, causing injuries that compromise plant development and photosynthetic capacity [4].

Using synthetic insecticides or genetically modified crop traits expressing *Bacillus thuringiensis* Berliner (Bt) toxin genes remains the primary approach for controlling *S. frugiperda*. However, these practices have raised concerns due to the increasing frequency of resistance. Selection pressure in the Brazilian agroecosystem is among the highest in the world, especially under irrigation, where *S. frugiperda* continuously finds host plants and is exposed to control strategies (chemical control and/or Bt crops) nearly year-round [5]. This accelerates the evolution of resistance in targeted populations.

Because steps to slow this evolutionary process in Brazil have not been implemented, resistance has at times rendered Bt crops or conventional insecticides ineffective within only a few insect generations. This loss of efficacy has led to increased use of synthetic molecules, intensifying environmental impacts and posing risks to human health [6]. Some DNA viruses, such as baculoviruses, infect insects and are used as biological control tools. These biological insecticides, including *S. frugiperda* multiple nucleopolyhedrovirus (SfMNPV), have a favorable ecotoxicological profile because they are non-toxic to non-target insects and can be incorporated into insect resistance management and integrated pest management programs [7].

Biological control has emerged as an effective tool for maintaining sustainability in agroecosystems and safeguarding environmental and human health [8]. Baculoviruses belong to the family Baculoviridae, which comprises large, circular, double-stranded DNA viruses [9]. They occur naturally in Lepidoptera and can be isolated and produced commercially. SfMNPV, a nucleopolyhedrovirus, is ingested by *S. frugiperda* larvae, within which it replicates and produces new viral particles. Infection causes severe damage and disruption to the insect’s cells and tissues, leading to progressive disease and low survival of infected larvae [10,11,12].

Another advantage of SfMNPV is its compatibility with other control strategies. For example, mixtures of SfMNPV with chemical pesticides can be used to manage *S. frugiperda* in maize [13,14], and there is evidence of an additive effect with the Cry1Ac insecticidal protein expressed in Bt soybean [15]. Given the increasing use of SfMNPV in large-scale crop production in Brazil [15], this study assessed its biological activity on a laboratory-maintained population of *S. frugiperda*. Based on evidence that sublethal infections can impose developmental costs [16], we hypothesized that applying low concentrations of the SfMNPV isolate from the commercial product Cartugen^®^ would not only reduce larval survival under laboratory conditions but also induce sublethal effects in surviving individuals, such as decreased leaf consumption, reduced body size, and lower growth rates.

## 2. Materials and Methods

The study was conducted at the Entomology Laboratory of the Department of Plant Science and Environmental Sciences, located at the Center of Agricultural Sciences of the Federal University of Paraíba (CCA/UFPB), Areia, PB. *Spodoptera frugiperda* larvae were obtained through laboratory rearing. The population was originally collected in a non-Bt cornfield and maintained in the laboratory for eighteen generations in Areia, Paraíba, Brazil (S 6°58′11.6″ and W 35°43′56.8″).

For rearing, the larvae were kept in glass tubes containing an artificial diet based on bean (240 g), wheat germ (120 g), brewer’s yeast (72 g), ascorbic acid (7.3 g), sorbic acid (2.4 g), methyl parahydroxybenzoate (Nipagin) (4.4 g), vitamin solution (10 mL), formaldehyde (40%), agar (20 g), and distilled water (1000 mL) [17]. Rearing took place in a controlled environment of 26 °C ± 1 °C, 60% ± 10% relative humidity, and a 12 h photophase. The larvae were kept in ordinary glass tubes without lids, filled with the artificial diet and placed vertically on tube racks [18]. A hydrophilic cotton ball was used to seal the tube end until pupation. Afterward, pupae were sexed and transferred to polyvinyl chloride cages measuring 12.5 × 12.5 × 20.0 cm, where they remained until adult emergence. Females laid egg masses, each containing 50–250 eggs, which were collected and placed in plastic containers measuring 11 × 7.5 cm.

### 2.1. Bioassays

The insects were exposed to the virus before being transferred to the plants. The experiment used a completely randomized design with different concentrations and 50 replicates (one larva per replicate). The following concentrations of baculovirus were used: 9.00 × 10^3^, 1.80 × 10^4^, 3.75 × 10^4^, 7.50 × 10^4^, 1.35 × 10^5^, 2.25 × 10^5^, and 3.75 × 10^5^ occlusion bodies (OB)/mL. Distilled water served as the control. We chose these concentrations based on a preliminary dose–response bioassay.

Solutions containing baculovirus were diluted in distilled water, green dye, and 10% and 5% sucrose, respectively, and then mixed by vortexing. Droplets were deposited into a 60 mm Petri dish. Each droplet was 0.5 μL in volume and dispensed using an electric pipette (Pipetman P10; Gilson, Middleton, WI, USA). Neonates (6–8 h after hatching) were released in the center of the Petri dish and allowed to feed on the solution for 15 min. Fifty neonates were placed in each dish, which was sealed with parafilm following Bentivenha et al. [19]. After 15 min, neonates showing green coloration in the thorax and abdomen (confirming ingestion) were carefully transferred to corn plants. Corn plants 15–25 days old were used. Plants without virus-containing solutions served as the control, and the larvae were individually placed in 770 mL plastic cups covered with voile fabric, secured with elastic bands to prevent escape.

To quantify OB/mL, two sets of the Cartugen^®^ sample were serially diluted in microtubes to a 200× dilution in Tween 80 (0.05% *v*/*v*). A volume of 9.4 μL of the diluted sample was applied to each side of a 0.1 mm–depth Bright-Line hemocytometer. The hemocytometer rested on a flat surface for 10 min to allow particles to settle. Under 400× magnification on a brightfield microscope, nine equally spaced 200- × 200-μm squares on the central grid were counted for a total number N of OBs on each side. The OB concentration was determined using the formula:

OBs/mL = (N/9) × 25 × 200 × 10,000

OB quantification was considered valid when the mean count of at least two dilution sets (four counts) had a standard deviation below 10%.

Assessments were made throughout all larval stages. The experiment was monitored daily for 12 days, until larvae reached the pupal stage. Dead insects were defined as individuals unable to walk on the leaves and showing color change upon death. At the end of the experiment, surviving larvae were weighed. The following variables were evaluated: larval survival, daily leaf consumption rate, head diameter, body length, body width, and weight at the end of the larval stage.

Leaf area consumed by the larvae was quantified from images taken before and after feeding. Photographs were taken with a wide-angle camera (equivalent to 13 mm) and a standard camera (equivalent to 24 mm). ImageJ Version 1.54p was used to measure leaf areas for each selected plant. The first step was calibrating the scale. Images needed to be clear, without erasures or objects at the margins, because the software interpreted any detail above the defined scale as damage. The Area, Perimeter, Feret’s diameter (a measurement along a specified direction), and Median options were selected to configure measurements. All data were recorded in an Excel file, and total area was compared to the consumed area.

### 2.2. Data Analysis

All analyses were conducted in R version 4.4.2 [20]. Survival analysis focused on the expected duration until a mortality event, with failure time considered after the pupal stage as a censored observation. The study of insect survival in the laboratory was carried out using the “survminer” and “survival” packages [21]. Nonparametric survival estimates were compared pairwise across groups, with multiple comparisons adjusted using the pairwise_survdiff function, which performs pairwise comparisons across group levels while adjusting for multiple comparisons.

We estimated leaf consumption rates at pathogenic concentrations, along with corresponding 95% confidence intervals (CIs). To calculate the CIs for leaf consumption rates and body size, we used the *lm()* function in R [20] to create a linear regression model. This function takes the R formula Y ~ X, where Y is the response variable (leaf consumption rate or body size) and X, the predictor variable, is the product concentration. The variables head diameter, body length, and body width were compared using contrasts generated by the Gaussian linear model.

The survival rate was also analyzed with a probit model. The estimate of the pathogenic concentration at 50% was obtained using the *LC_probit* function from the “ecotox” package, with 95% fiducial confidence limits applied to the selected lethal concentration (LC). *LC_probit* estimates the LC and its fiducial confidence limits via probit analysis, returning a data frame that includes predicted LC values at the 95% level and key parameters, such as fiducial limits and slope. Data on the mean weight of larvae that survived exposure to the virus were analyzed using nonlinear regression to estimate the effective concentration 50% (EC_50_), defined as the baculovirus concentration in the insect body that produces a defined degree of weight inhibition, along with its 95% CI. The nonlinear model used to calculate weight inhibition was [17]:

(1) weight = W_0_/[1 + (concentration/EC_50_)^B^]

where B is a constant and W_0_ is the estimated weight for the control treatment.

## 3. Results

**Figure 1:**
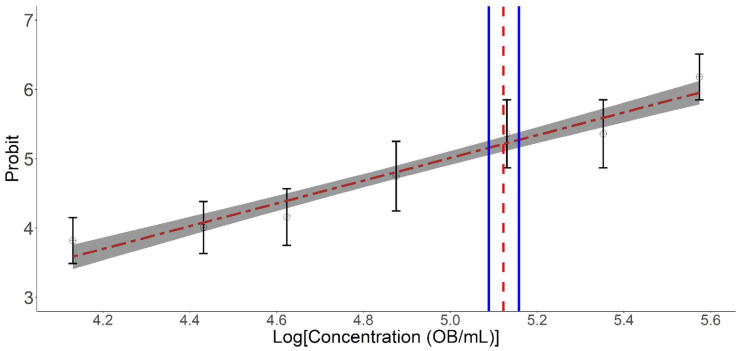
Concentration response of OBs of SfMNPV from the commercial product Cartugen^®^ on *S. frugiperda* larvae. Points = observed data; Bars = standard error; Dashed line = response predicted by the probit model. The vertical red line is the median LC (LC_50_). Blue vertical lines represent the 95% CI associated with the LC_50_.

The results obtained in this study reveal a linear decrease in survival (probit model; χ^2^ = 36.43, df = 5, *p* = 0.0010) in *S. frugiperda* larvae as a function of the logarithmically adopted concentrations of the Cartugen^®^ product (Figure 1). The following parameters best describe this pattern: a slope of 0.533 ± 0.102 and an intercept of −2.35 ± 1.99. The concentration of 3.75 × 10^5^ OBs/mL yielded the lowest observed survival (approximately 10%), which differed significantly from the other concentrations (Figure 1).

**Figure 2:**
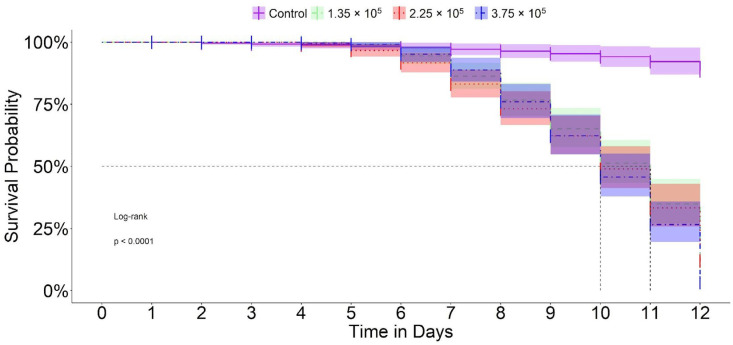
Survival pattern of *S. frugiperda* as a function of different spray concentrations of OBs of SfMNPV from the commercial product Cartugen^®^.

Based on the Cartugen^®^ concentrations evaluated, acute infection in second-instar *S. frugiperda* larvae was confirmed. When the larvae were sprayed, the LC_50_ was estimated at 1.32 × 10^5^ OBs/mL (95% CI = 1.22 × 10^5^–1.43 × 10^5^ OBs/mL) (Figure 1). Using only the control and concentrations that promoted ≤50% survival, we found that the median lethal time—the time required for 50% of the larvae to die—ranged from 10 to 11 days following baculovirus ingestion (Figure 2).

### Weight Gain Inhibition

The results of this study revealed a clear pattern of weight gain inhibition in *S. frugiperda* as a function of SfMNPV concentration. The estimated effective concentration (EC_50_) of the virus was 1.16 × 10^4^ OB/mL (95% CI = 1.07 × 10^4^–1.24 × 10^4^ OBs/mL), indicating that even low concentrations of the baculovirus can reduce the average weight of surviving larvae by 50%. The expected weight in the control (W_0_) was estimated at 47.40 mg (95% CI = 46.50–48.40 mg). The slope parameter B was estimated to be 1.42, indicating that the degree of weight inhibition increased with concentration (Table 1).

**Figure 3:**
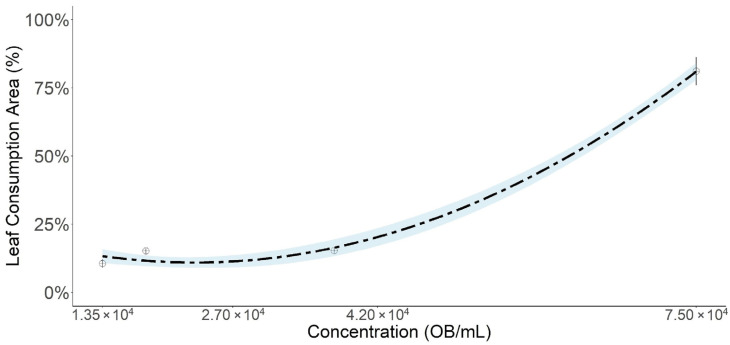
Leaf consumption (%) expressed as the ratio between consumed leaf area and total leaf area offered, at different concentrations of OBs of SfMNPV from the commercial product Cartugen^®^ in *S. frugiperda* larvae. Points = observed data; Bars = standard error; Dashed line = response predicted by the normal model with inverse link function.

The baculovirus concentrations analyzed also significantly affected leaf consumption, with inhibition at lower concentrations and increased consumption at higher concentrations among surviving larvae (Figure 3). The polynomial quadratic regression equation to predict the leaf consumption expressed as the ratio between consumed leaf area and total leaf area offered, at different concentrations of OBs of SfMNPV was estimated in −1.01 + 2.73x + 0.84x^2^ (R^2^ = 0.84; *p* < 0.01).

When exposed to baculovirus, the head diameter, body length, and body width of *S. frugiperda* varied from 0.32 mm (4.20 × 10^4^ OBs/mL) to 0.44 mm (1.35 × 10^4^ OBs/mL), from 3.61 mm (4.20 × 10^4^ OBs/mL) to 6.67 mm (1.35 × 10^4^ OBs/mL), and from 0.34 mm (4.20 × 10^4^ OBs/mL) to 0.73 mm (1.35 × 10^4^ OBs/mL), respectively. Although these values did not differ significantly among the concentrations tested, all biometric parameters were lower than those observed in the control (Table 2).

## 4. Discussion

The survival pattern of *S. frugiperda* was proportional to the applied concentration, with approximately 10% survival at the maximum concentration of 3.75 × 10^5^ OB/mL, indicating that differences in survival among treatments were mainly due to a shorter time to mortality. The larval weight pattern followed the same trend, decreasing as viral concentration increased. Although a stimulatory effect on *S. frugiperda* larvae could be expected at some OB concentrations—where low viral doses do not kill the larvae and allow continued development [22]—our data revealed an inhibitory effect on leaf consumption at the lowest concentrations tested and a stimulatory effect only at higher concentrations.

Overall, we observed that baculovirus not only reduced survival in *S. frugiperda* but also altered several biometric variables. Because baculoviruses primarily infect larvae during early developmental stages through ingestion, these biometric effects reflect the progression of infection. Once ingested, OBs release virions that bind to midgut receptors and enter epithelial cells, spreading via the hemolymph and ultimately causing systemic infection and cell death [7,23].

The survival time of larvae exposed to baculovirus has been widely investigated. Our findings are consistent with previous studies showing that higher bioinsecticide concentrations lead to greater OB uptake, increased virulence, and, consequently, shorter median lethal times, thereby reducing the lethal concentration required [24]. The longer median lethal times observed at the lowest concentrations in our experiment may be related to sublethal effects, low viral load, or partial degradation or inactivation of viral particles—factors that can create small fluctuations in infection intensity. Notably, the lowest concentrations resulted in reduced feeding and developmental rates, which could be advantageous for managing subsequent pest generations [25]. When virus concentrations decline, the insect’s defense system gains a temporary advantage, and the midgut can function as a protective barrier. This phenomenon, known as hormesis, describes how low viral doses may fail to cause mortality due to enhanced detoxification or immune responses [26,27,28].

The estimated effective concentration for reducing larval weight by 50% (EC_50_ = 1.16 × 10^4^ OBs/mL) indicates that survival and sublethal impacts occur even at relatively low viral concentrations. The slope parameter from the nonlinear regression about larval weight also confirmed that weight inhibition increased with concentration. These findings suggest that exposure to low viral doses reduces larval metabolic activity. As baculoviruses have coevolved with their hosts, they have developed mechanisms to enhance their fitness during infection [29]. For instance, leaf-feeding rates increased with higher concentrations of SfMNPV, and, in some cases, viruses can manipulate host physiology or behavior to facilitate viral transmission.

Broadly, low concentrations of baculovirus reduced leaf-feeding rate, average larval weight, and the biometric measurements (head diameter, body length, and body width). Baculovirus infection alters host behavior and physiology, affecting mobility and feeding. These changes are often regulated by viral genes that modulate host signaling pathways in the central nervous system. As reported by Llopis-Giménez et al. [29], baculovirus infection reduced larval weight by approximately 25% and fecal mass by 50% in *S. exigua*, likely due to altered neuropeptide expression, including overexpression of a proctolin-like peptide. Infection may also impair chemoreception by altering odorant receptor gene expression. Llopis-Giménez et al. [29] showed that infected *S. exigua* larvae altered expression of SexiOR23 and SexiOR35, reducing responsiveness to plant volatiles and impairing food-source localization. Together, digestive disruptions, hormonal imbalance, and altered odorant receptor expression contribute to the increased susceptibility of *S. frugiperda* to SfMNPV.

## 5. Conclusions

SfMNPV affects the growth of the fall armyworm and alters its feeding rate. Our analysis shows that the impact of SfMNPV extends beyond mortality: even low concentrations can influence larval development in *S. frugiperda*. Despite the promising results presented here, future field studies are essential—particularly those examining interactions between baculovirus and other bioinsecticides, as well as how environmental conditions and emerging technologies may influence viral effectiveness. Additional research is needed to better understand the plant–insect–virus relationship and to refine the integration of baculovirus-based products into pest management programs.

## Figures and Tables

**Table 1:** Nonlinear regression model for estimating EC_50_ parameters for *S. frugiperda* according to concentrations of OBs of SfMNPV from the commercial product Cartugen^®^.

Parameter	Estimate	Standard Error	95% CI
W_0_	47.40 mg	49.18	46.50–48.40 mg
B	1.42	0.07	1.28–1.57
EC_50_ (OBs/mL)	1.16 × 10^4^	4.27 × 10^2^	1.07 × 10^4^–1.24 × 10^4^

W_0_ = expected weight of the control; B = logistic function angular parameter; EC_50_ = effective concentration capable of reducing the larval weight by 50%.

**Table 2:** Biometric parameters of *S. frugiperda* exposed to concentrations of SfMNPV.

Concentration (OBs/mL)	Biological Parameters		
	Head Diameter (mm)	Body Length (mm)	Body Width (mm)
0	1.26 ± 0.05 (1.12–1.32) a	12.01 ± 0.31 (08.01–14.00) a	1.52 ± 0.20 (1.22–1.84) a
1.35 × 10^4^	0.44 ± 0.20 (0.18–0.77) b	6.67 ± 2.77 (2.82–10.52) b	0.73 ± 0.32 (0.27–1.18) b
2.70 × 10^4^	0.33 ± 0.22 (0.17–0.68) b	4.04 ± 2.40 (0.52–7.30) b	0.43 ± 0.28 (0.16–0.96) b
4.20 × 10^4^	0.32 ± 0.15 (0.12–0.55) b	3.61 ± 2.01 (0.90–6.40) b	0.34 ± 0.24 (0.14–0.64) b

Data are presented as mean ± standard error (95% CI). Means followed by the same letter do not differ according to contrasts generated by the Gaussian linear model (*p* = 0.05).

## Data Availability

The original contributions presented in this study are included in the article. Further inquiries can be directed to the corresponding author.
